# Screening of selective histone deacetylase inhibitors by proteochemometric modeling

**DOI:** 10.1186/1471-2105-13-212

**Published:** 2012-08-22

**Authors:** Dingfeng Wu, Qi Huang, Yida Zhang, Qingchen Zhang, Qi Liu, Jun Gao, Zhiwei Cao, Ruixin Zhu

**Affiliations:** 1 School of Life Sciences and Technology, Tongji University, Shanghai, 200092, P.R. China; 2 School of Information Engineering, Shanghai Maritime University, Shanghai, 201306, P.R. China; 3 Institute for Advanced Study of Translational Medicine, Tongji University, Shanghai, 200092, P.R. China; 4 School of Pharmacy, Liaoning University of Traditional Chinese Medicine, Dalian, Liaoning, 116600, P.R. China

**Keywords:** Histone deacetylases inhibitors, Proteochemometric, Selective inhibitors

## Abstract

**Background:**

Histone deacetylase (HDAC) is a novel target for the treatment of cancer and it can be classified into three classes, i.e., classes I, II, and IV. The inhibitors selectively targeting individual HDAC have been proved to be the better candidate antitumor drugs. To screen selective HDAC inhibitors, several proteochemometric (PCM) models based on different combinations of three kinds of protein descriptors, two kinds of ligand descriptors and multiplication cross-terms were constructed in our study.

**Results:**

The results show that structure similarity descriptors are better than sequence similarity descriptors and geometry descriptors in the leftacterization of HDACs. Furthermore, the predictive ability was not improved by introducing the cross-terms in our models. Finally, a best PCM model based on protein structure similarity descriptors and 32-dimensional general descriptors was derived (R^2^ = 0.9897, Q_test_^2^ = 0.7542), which shows a powerful ability to screen selective HDAC inhibitors.

**Conclusions:**

Our best model not only predict the activities of inhibitors for each HDAC isoform, but also screen and distinguish class-selective inhibitors and even more isoform-selective inhibitors, thus it provides a potential way to discover or design novel candidate antitumor drugs with reduced side effect.

## Background

All over the world, tumor is the second incurable disease only to cardiovascular disease. A wide range of proteins are found to be related to tumor formation and metastasis. However, only proteins with widespread biological significance for the tumor cells growth regulation are most possible to be the targets of broad-spectrum low-toxic antitumor drugs. In recent studies, histone deacetylases (HDACs) are proved to be novel epigenetic targets for the treatment of cancer [1-3]. Histone deacetylase inhibitors (HDACi) have extensively demonstrated the antitumor efficacy *in vitro* and *in vivo*. Therefore, the related study of HDACi has become one of the most important research fields of the antitumor drugs, especially during the coming area of epigenetics.

Histone deacetylases comprise a superfamily of 18 genes which is divided into two families and four classes in eukaryotic cells. Classes I, II, and IV consist of 11 family members, which are referred to as “classical” HDACs, whereas the 7 class III members are called “sirtuins” [4].Classical HDACs which require Zn^2+^ as a cofactor for their deacetylase activity are a promising novel class of anti-cancer drug targets that can be inhibited by Zn^2+^ chelating compounds such as hydroxamic acids. In contrast, these compounds are not active against sirtuins as these class III enzymes have a different mechanism of action in requiring NAD^+^ as an essential cofactor [5]. Recent researches indicate that sirtuins are linked to aging as well as metabolic and neurodegenerative diseases [6].

Classical HDACs are classified based on their homology to yeast proteins. HDACs 1, 2, 3, and 8 which belong to Class I have homologies to yeast RPD3, and they are located within the nucleus. HDACs 4, 5, 6, 7, 9, and 10 which belong to Class II have homologies to yeast HDA1 and located in both the nucleus and the cytoplasm. It should be noted that Class II HDACs can be further subdivided based on their sequence homolog and domain organization, *i.e.* Class IIa, which include HDACs 4, 5, 7, and 9 containing an N-terminal extension with regulatory function, and Class IIb, which include HDACs 6 and 10 containing two catalytic domains. HDAC 11 is categorized into class IV with conserved residues in its catalytic center that are shared by both classes I and II HDACs. The classification of classical HDACs is summarized in Table 1.

**Table 1:** “classical” HDACs

**HDAC**		**Localization**	**Chromosomal site**	**References**
Class I (RPD3 homologue)				
	HDAC1	Nucleus	1p34.1	[7]
	HDAC2	Nucleus	6p21	[8]
	HDAC3	Nucleus	5p31	[9]
	HDAC8	Nucleus	Xq13	[10]
Class II (HDA1 homologue)				
IIa	HDAC4	Nuc/Cyt	2q372	[11]
	HDAC5	Nuc/Cyt	17q21	[12]
	HDAC7	Nuc/Cyt	12q13	[13]
	HDAC9	Nuc/Cyt	7p21-p15	[14]
IIb	HDAC6	Mainly Cyt	Xp11.22-33	[15]
	HDAC10	Mainly Cyt	22q13.31-33	[16]
Class IV				
	HDAC11	Nuc/Cyt	3p25.2	[17]

Histone deacetylase inhibitors (HDACi) that act on 11 zinc-dependent HDAC isozymes generally possess a zinc-binding group which coordinates the zinc ion in the active site, a cap substructure that interacts with amino acids at the entrance of the N-acetylated lysine binding channel, and a linker connecting the cap and the zinc-binding group at a proper distance [18]. HDACi can be categorized into four subtypes based on their chemical structures: (1) short chain fatty acid; (2) hydroxamic acid; (3) benzamides; and (4) cyclic peptides. Since HDACi do not inhibit all HDAC isoforms to the same extent, they can be categorized into pan-HDAC inhibitors and selective HDAC inhibitors including class I-specific inhibitors, class II-specific inhibitors, and class IV-specific inhibitors. Currently, many HDAC inhibitors have already been tested in clinical trials and shown certain antitumor or other biological activity. However, some HDAC inhibitors, especially pan-inhibitors, indicate serious side effects, such as fatigue, nausea, anorexia, diarrhea, thrombus formation, thrombocytopenia, neutropenia, anemia, myalgia, hypokalemia, hypophosphatemia, *etc.*[3]. Thus, HDAC inhibitors are possible to greatly improve the efficacy and reduce the certain toxicities only when they target the most relevant HDAC isoform rather than multiple ones. Consequently, it should be useful to discover or design novel antitumor drugs with fewer side effects when one method can analyze the interaction of inhibitors against multiple HDACs with further sorting out isoform- or class-specific inhibitors.

As for *in silico* drug discovery, there are many methods available such as molecular docking [19,20], pharmacophore models, quantitative structure-activity relationship (QSAR) [21-23], protein-ligand interaction fingerprint-based screening [24,25] and others [26-29]. QSAR is a widely applied computational method for predicting chemical compounds’ interactions with a single target protein. However, when thousands of chemical compounds interacted with 11 different HDAC isoforms, 11 separate QSAR models for each HDAC isoform are needed to create, which is quite complicated and time consuming. In addition, these separate models cannot extended to predict inhibitions of new HDACs [30]. Therefore, a new method should be proposed to predict cross-interactions of chemical compounds to multi-HDAC isoforms simultaneously.

More recently, proteochemometric (PCM) modeling has been widely used to study the cross-interactions between a series of compounds and a series of proteins. In this area Maris Lapinsh et.al studied melanocortin chimeric receptors using partial least-squares projections (PLS) to deduce PCM models [31,32]; Hanna Geppert et.al derived PCM models of eleven proteases from four different protease families by support vector machine [33]; Ilona Mandrika and Maris Lapinsh et.al applied PLS to model interactions of HIV mutants [30,34] and antibodies [35]. Contrary to traditional QSAR, PCM is based on the similarity of a group of ligands together with that of a group of targets [36]. Consequently, PCM can integrate several separate QSAR models into a global one. With the global PCM model in hand, we can study the cross interactions of all the ligands with all the targets in the data set or even outside the data set. By predicting the affinity for each ligand-target pair, PCM models can describe the specific interaction between a ligand and a target and discriminate the interaction strength between different ligand-target pairs. Therefore, in our study PCM models were applied to study the cross-interactions of a series of HDAC inhibitors to five HDAC isoforms, *i.e.*, HDAC2, HDAC4, HDAC6, HDAC7, and HDAC8.

## Results and discussion

### Proteochemometric modeling

In our study, 18 proteochemometric models were created from training set with combinations of different descriptors. As shown in Table 2, goodness-of-fits (R^2^s) of all models were higher than 0.9619 and their cross validation coefficients Q_cv_^2^ ranged from 0.5734 to 0.7162. The model derived based on P1 and GD showed to be the best model with the highest predictive ability (Q_cv_^2^ = 0.7162 and Q_test_^2^ = 0.7542). Accordingly P1-GD model was used in the subsequent analysis.

**Table 2:** **Goodness-of-fit (R**^**2**^**) and predictive ability (Q**^**2**^_**cv**_**, Q**^**2**^_**test**_**) of the obtained 18 models**

**Model**	**R**^**2**^	**Q**^**2**^_**cv**_	**Q**^**2**^_**test**_	**Model**	**R**^**2**^	**Q**^**2**^_**cv**_	**Q**^**2**^_**test**_
P0-DLI^a^	0.9616	0.6564	0.7292	P1-GD-C^b^	0.9909	0.6732	0.7191
P0-GD	0.9895	0.6960	0.7331	P2-DLI-C	0.9883	0.6264	0.6519
P1-DLI	0.9619	0.6757	0.7427	P2-GD-C	0.9917	0.5734	0.6215
P1-GD	0.9897	0.7162	0.7542	C(P0,DLI)^c^	0.9860	0.6484	0.6941
P2-DLI	0.9614	0.6521	0.7272	C(P0,GD)	0.9914	0.6302	0.6772
P2-GD	0.9894	0.6858	0.7268	C(P1,DLI)	0.9811	0.6686	0.7227
P0-DLI-C	0.9871	0.6462	0.6944	C(P1,GD)	0.9904	0.6625	0.7190
P0-GD-C	0.9916	0.6319	0.6759	C(P2,DLI)	0.9898	0.6505	0.6067
P1-DLI-C	0.9846	0.6626	0.7251	C(P2,GD)	0.9941	0.5967	0.5597

^a^ P0-DLI means this model is based on protein descriptor P0 and ligand descriptor DLI.

^b^ P1-GD-C means this model is based on protein descriptor P1, ligand descriptor GD and cross-terms.

^c^ C(P0,DLI) means this model is based on only cross-terms of P0 and DLI.

### P0 vs P1 vs P2

Three protein descriptors, *i.e.*, sequence similarity descriptor (P0), structure similarity descriptor (P1) and geometry descriptor (P2), were used to describe HDACs in our study. Sequence similarity descriptor is based on the sequence identities of HDACs, while structure similarity descriptor and geometry descriptor leftacterize HDACs based on their 3D-structures. Protein descriptors are different from ligand descriptors since proteins have larger molecule structures to describe. If available, proteins are likely to be described on the basis of crystal structures. Protein structure similarity descriptor was calculated by protein 3D-struture alignment with more sufficient information considered. Contrary to P1, P0 only leftacterizes protein based on sequence alignment, and may lose certain 3D information of proteins. Not surprisingly, models derived from P1 showed a better predictive ability than those of P0 (Table 3). In addition, although P2 is also derived based on 3D-structure, it only measures bond length, bond angle and dihedral angle statistically without much of the detailed information of proteins, thus it is not sufficient to leftacterize proteins comprehensively. As a result, we also found that models based on geometry descriptor obtained the worst predictive ability (Q_test_^2^ of models based on P2 in every group is the lowest) compared to the others.

**Table 3:** **R**^**2 **^**and Q**^**2**^_**test**_**of 18 models grouped for comparing three protein descriptors ability**

**Model**	**R**^**2**^	**Q**^**2**^_**test**_	**Model**	**R**^**2**^	**Q**^**2**^_**test**_
Group 1			Group 2		
P0-DLI	0.9616	0.7292	P0-GD-C	0.9916	0.6759
P1-DLI	0.9619	**0.7427**	P1-GD-C	0.9909	**0.7191**
P2-DLI	0.9614	0.7272	P2-GD-C	0.9917	0.6215
Group 3			Group 4		
P0-GD	0.9895	0.7331	C(P0,DLI)	0.9860	0.6941
P1-GD	0.9897	**0.7542**	C(P1,DLI)	0.9811	**0.7227**
P2-GD	0.9894	0.7268	C(P2,DLI)	0.9898	0.6067
Group 5			Group 6		
P0-DLI-C	0.9871	0.6944	C(P0,GD)	0.9914	0.6772
P1-DLI-C	0.9846	**0.7251**	C(P1,GD)	0.9904	**0.7190**
P2-DLI-C	0.9883	0.6519	C(P2,GD)	0.9941	0.5597

The highest Q_test_^2^s of every group are highlighted.

### GD vs DLI

Similar to protein descriptors, two typical kinds of ligand descriptors, *i.e.*, General Descriptor (GD) and Drug-Like Index (DLI) were applied. Our result indicates that there was no significant difference between Q^2^ values of models based on GD and DLI **(**Table 4), with p-value bigger than 0.1 by paired *t*-test.

**Table 4:** **Q**^**2 **^_**test **_**of 18 models grouped by ligand descriptors**

**X**	**P0-X**	**P1-X**	**P2-X**	**P0-X-C**	**P1-X-C**	**P2-X-C**	**C(P0,X)**	**C(P1,X)**	**P(P2,X)**
GD	0.7331	0.7542	0.7268	0.6759	0.7191	0.6215	0.6772	0.7190	0.5597
DLI	0.7292	0.7427	0.7272	0.6944	0.7251	0.6519	0.6941	0.7227	0.6067

Paired t test: t = 1.746.

It should be noted that there are a large number of different descriptors available for ligands, and there is no optimal one suitable for all the data sets. Therefore, it is wise to try several different descriptors to identify the optimal one in a particular scenario [37]. In our study, we used two different ligand descriptors, GD and DLI to create PCM models. These two kinds of descriptors leftacterize physical properties and topological indices of ligands respectively. For our particular data set, there was no statistically difference in predictive ability between these two ligand descriptors.

### Model performance with or without cross-terms

A multiplied cross-term was used in our models and it was shown to be helpless in the improvement of the predictive ability of PCM models. The Q_test_^2^ of models with cross-terms is lower than that without cross-terms in every group (Table 5).

**Table 5:** **R**^**2**^**and Q**^**2**^_**test**_**of 12 models grouped by with- or without- cross-terms**

**Model**	**R**^**2**^	**Q**^**2**^_**test**_	**Model**	**R**^**2**^	**Q**^**2**^_**test**_
Group 1			Group 2		
P0-DLI	0.9616	**0.7292**	P1-GD	0.9897	**0.7542**
P0-DLI-C	0.9871	0.6944	P1-GD-C	0.9909	0.7191
Group 3			Group 4		
P0-GD	0.9895	**0.7331**	P2-DLI	0.9614	**0.7272**
P0-GD-C	0.9916	0.6759	P2-DLI-C	0.9883	0.6519
Group 5		Group 6			
P1-DLI	0.9619	**0.7427**	P2-GD	0.9894	**0.7268**
P1-DLI-C	0.9846	0.7251	P2-GD-C	0.9917	0.6215

The highest Q_test_^2^s of every group are highlighted.

Although cross-terms are intended to describe the properties of the interface between ligand and protein, there is still no good descriptor for the representation of local receptor-ligand interfaces [37], which may possibly result in the worse performance of the multiplied cross-term in our PCM models. Recently, a new Protein-Ligand interaction fingerprint was derived for *in silico* screening [24,25]. This interaction fingerprint is a local descriptor to represent the interfaces of receptor-ligand and proved to be a good candidate cross-term in PCM. Theoretically, it should achieve better performance if the crystal complex structure exists. However, since there is no crystal structure available for most of the receptor-ligand pairs in our data set, thousands of complex structures have to be produced by molecular docking to apply interaction fingerprint, which may result in biases. Therefore, the interaction fingerprint was not adopted in our study.

### Selective ability of proteochemometric model

In our study, we aimed to exploit an effective method to screen selective HDAC inhibitors which has selective activity on a single or a specific class of HDAC isoforms. For this purpose, proteochemometrics was applied to analyze the interaction strength of inhibitors against multiple HDACs, and then select out isoform-specific, class-specific as well as pan inhibitors. To verify the performance of the derived PCM models, an external validation of ten inhibitors was carried out to predict affinity with the best model (P1-GD model). The predicted values are compared with the corresponding experimental ones as shown in Table 6.

**Table 6:** **The activity data and P0-GD model predict affinity data of ten HDAC inhibitors**^**a**^

**Class I**		**Class IIa**		**Class IIb**
**HDAC2**	**HDAC8**	**HDAC4**	**HDAC7**	**HDAC6**
Pan-HDAC inhibitors				
TSA				
**S**	**W**	**S**	**S**	**S**
−0.720	***0.342***	***1.027***	***0.660***	***0.087***
SAHA				
**S**	**W**	**S**	**S**	**S**
−0.464	−1.092	***0.687***	***1.031***	−0.094
Panbinostat(LBH589)				
**S**	**W**	**S**	**S**	**W**
***0.742***	***0.391***	***0.524***	***0.347***	***0.996***
Belinostat(PXD-101)				
**S**	**W**	**S**	**S**	**S**
***0.327***	−0.330	***1.183***	***0.643***	***1.339***
Class I-specific inhibitors				
MGCD0103				
**S**	N	N	N	N
***0.296***	−0.946	−0.557	−1.018	−0.963
depsipeptide(FK228)				
**S**	nd	**W**	nd	N
***0.954***	−0.095	***0.687***	***0.438***	−0.167
Apicidin				
**S**	**W**	N	N	N
***0.238***	***0.096***	−0.501	−0.176	−0.120
Class II-specific inhibitors				
APHA				
nd	nd	**S**	nd	nd
−0.196	−0.089	−0.204	−0.194	***0.271***
Tubacin				
nd	nd	nd	nd	**S**
***0.148***	−0.687	−0.293	−0.301	***1.414***
NCT-10a				
nd	nd	**W**	nd	**S**
−0.405	−0.731	***0.137***	***0.159***	***0.010***

^a^ S, W,N and nd is the experimental affinity of inhibitors and the numerical number is the predicted affinity data. Those predicted values larger than 0 are supposed to have inhibition with highlighted.

S strong inhibition.

W weak inhibition.

N no inhibition.

nd no data pubilshed.

Among the ten inhibitors for external validation, TSA, SAHA, LBH589 and PXD-101 are reported as pan-HDAC inhibitors and almost all their predicted affinity values are high for all the HDAC isoforms in our test (e.g. LBH HDAC2 0.742, HDAC8 0.391, HDAC4 0.524, HDAC7 0.347, HDAC6 0.996). In addition, MGCD0103, FK228 and Apicidin are reported as class I-specific inhibitors and our results also indicated that the predicted values for class I HDACs are higher than those of others (e.g. Apicidin HDAC2 0.238, HDAC8 0.096, HDAC4 -0.501, HDAC7 -0.176, HDAC6 -0.120). Finally APHA, Tubacin and NCT-10a are reported as class II-specific inhibitors and our results are consistent with the validation data that their predicted values are higher for class II HDACs (e.g. NCT-10a HDAC2 -0.405, HDAC8 -0.731, HDAC4 0.137, HDAC7 0.159, HDAC6 0.010).

As a conclusion, our best PCM model performs well in screening selective HDAC inhibitors and distinguishing pan-HDAC inhibitors, class I-specific inhibitors and class II-specific inhibitors successfully. Therefore, this model can be further used to screen class-selective inhibitors as well as isoform-selective inhibitors of HDACs with fewer side effects.

## Conclusion

Although more and more HDAC inhibitors have been identified to date, the number of class-selective inhibitors or isoform-selective inhibitors is insufficient. Thus, it is important to find these selective inhibitors which are candidate therapeutic agents for tumor with reduced side effects. In this study, proteochemometric models were derived to analyze the inhibitory activity of 1275 compounds with 5 HDAC isoforms simultaneously. Among these models, the best one, P1-GD model, was highly predictive (Q_test_^2^ = 0.7542) and presented powerful ability to distinguish selective HDAC inhibitors from the pan ones. As a conclusion, proteochemometric modeling proves to be a suitable methodology for the prediction of inhibitor interactions with HDAC isoforms. Our study also indicates that the obtained optimal model can be potentially used for designing candidate antitumor drugs which can selectively target on a single HDAC or a specific class of HDAC isoforms.

## Methods

### Data set

To describe proteins more efficiently, five HDAC isoforms with known crystal structures were selected (Table 7). Among these isoforms, HDAC2 and HDAC8 are Class I HDACs; HDAC4, HDAC6, and HDAC7 are Class II HDACs, and more specifically, HDAC4 and HDAC7 belong to Class IIa; HDAC6 belongs to Class IIb.

**Table 7:** HDACs’ sequences and 3D structures from NCBI and PDB

**Protein**	**PDB entry**	**NCBI entry**	**Length(aa)**	**Class**
HDAC2	3MAX	NP_001518	488	I
HDAC4	2VQJ	NP_006028	1084	IIa
HDAC6	3C5K	NP_006035	1215	IIb
HDAC7	3C0Z	NP_056216	991	IIa
HDAC8	1 T69	NP_060956	377	I
HDAC1	nd^a^	NP_004955	482	I
HDAC3	nd	NP_003874	428	I
HDAC5	nd	NP_005465	1122	IIa
HDAC9	nd	NP_478056	1011	IIa
HDAC10	nd	NP_114408	669	IIb
HDAC11	nd	NP_079103	347	IV

^a^ nd means no data published.

The half maximal inhibitory concentration (IC50) values for 1443 chemical compounds (Additional file 1: Table S4) interacting with these HDAC isoforms were collected from the Binding Database (BindingDB, http://www.bindingdb.org). After filtration, the data set was reduced to 1275 compound-HDAC pairs with IC50 values, and it contained 215 pairs for HDAC2, 197 for HDAC4, 531 for HDAC6, 46 for HDAC7, and 286 for HDAC8 respectively (Table 8).

**Table 8:** The distribution of binding affinity IC50 data

**Type**	**Total**	**Training set**	**Test set**
HDAC2/ligands	215	139	76
HDAC4/ligands	197	128	69
HDAC6/ligands	531	345	186
HDAC7/ligands	46	29	17
HDAC8/ligands	286	186	100
Total	1275	827	448

The distribution of data set for every HDAC isoform is unbalanced. Therefore, we divided the data set into a training set (65%) and a test set (35%) by stratified sampling [38] (Additional file 2: Table S1, Additional file 3: Table S2).

### Description of proteins

Three different sets of descriptors were used to leftacterize the five HDAC isoforms, *i.e.* sequence similarity descriptor (P0) [32], structure similarity descriptor (P1) and geometry descriptor (P2).

#### Sequence similarity descriptor

The amino acid sequences of all the HDACs were retrieved from NCBI (the entries are listed in Table 7**)**. EMBOSS [39,40] was used to calculate sequence identities of the five selected HDAC isoforms with all the HDAC isoforms. Finally we obtained 11 sequence similarity descriptors (Table 9).

**Table 9:** 11 sequence similarity descriptors of HDAC2, 4, 6, 7 and 8

	**Class I**				**Class IIa**				**Class IIb**		**Class IV**
	**HDAC1**	**HDAC2**	**HDAC3**	**HDAC8**	**HDAC4**	**HDAC5**	**HDAC7**	**HDAC9**	**HDAC6**	**HDAC10**	**HDAC11**
Class I											
HDAC2	85.1	100.0	51.9	30.7	10.2	9.6	9.7	9.9	9.4	14.2	18.9
HDAC8	30.8	30.7	34.4	100.0	9.0	8.4	9.9	10.8	8.1	13.6	21.4
Class IIa											
HDAC4	11.1	10.2	9.5	9.0	100.0	58.4	46.9	54.3	20.6	11.1	8.6
HDAC7	10.0	9.7	10.5	9.9	46.9	40.5	100.0	39.7	19.1	13.0	9.6
Class IIb											
HDAC6	9.5	9.4	7.7	8.1	20.6	17.2	19.1	16.6	100.0	23.4	7.4

For all possible HDACs pairs, sequence identities (in %) are reported.

#### Structure similarity descriptor

This descriptor extends protein sequence alignment to structure alignment based on sequence similarity descriptor. By pairwise structure alignment using Protein Comparison Tool [41], we calculated pairwise structure identities of the five selected proteins and obtained five descriptors (Table 10).

**Table 10:** Five protein structure similarity descriptors of HDAC2, 4, 6, 7 and 8

	**Class I**		**Class IIa**		**Class IIb**
	**HDAC2**	**HDAC8**	**HDAC4**	**HDAC7**	**HDAC6**
Class I					
HDAC2	1.000	0.407	0.182	0.182	0.031
HDAC8	0.407	1.000	0.180	0.186	0.048
Class IIa					
HDAC4	0.182	0.180	1.000	0.706	0.027
HDAC7	0.182	0.186	0.706	1.000	0.036
Class IIb					
HDAC6	0.031	0.048	0.027	0.036	1.000

#### Geometry descriptor

Protein contains various bonds like C-N, C-O, C-N-CA, and CA-C-O *etc*. By measuring the various bond length, bond angle and dihedral angle [42], 30 protein Geometry descriptors were obtained for each HDAC protein (Additional file 4: Table S3).

### Description of inhibitors

In our study, the HDAC inhibitors were represented by two kinds of feature space, *i.e.* 32-dimensional General Descriptors (GD) and 28-dimensional Drug-Like Index (DLI). These descriptors are widely applied to the construction of QSAR models. For general descriptors, they include atomic contributions to van der waals surface area, log P (octanol/water), molar refractivity, and partial leftge. GD leftacterize physical properties and describe organic compounds in boiling point, vapor pressure, free energy of salvation in water, solubility in water, thrombin/trysin/factor Xa activity, blood–brain barrier permeability, and compound classification *etc*. [43]. On the other hand, DLI leftacterize simple topological indices of compounds and measure the hierarchy of drug structures in terms of rings, links, and molecular frameworks [44].

### Protein-inhibitor cross-terms

Evidently, ligand-receptor recognition can only be partially explained by linear combinations of ligand and receptor descriptors. In reality, protein-ligand interactions are governed by complex processes that depend on the complementarity of the properties of the interacting entities. In PCM, this is accounted for by protein-inhibitor cross-terms [31,36], which in the simplest case is obtained by multiplication of mean centered descriptors of proteins and inhibitors. Therefore, we obtained 11 × 32 = 352, 5 × 32 = 160, 30 × 32 = 960, 11 × 28 = 308, 5 × 28 = 140, 30 × 28 = 840 cross-terms for P0-GD, P1-GD, P2-GD, P0-DLI, P1-DLI, and P2-DLI respectively.

### Preprocessing of data

To reduce the bias of the model, all descriptors were mean centered and scaled to unit variance prior to the calculation of protein-ligand cross-terms. Moreover, the binding affinities (IC50) were logarithmically transformed to pIC50 and then mean centered and scaled to unit variance.

### Proteochemometric modeling

Support vector machine (SVM) is a non-linear modeling technique applied multiple times in PCM [33,45-50]. We created PCM models using support vector regression (SVR) built in Weka suit (Weka implementation “SMOreg”). Eighteen different combinations of descriptor blocks were constructed to derive PCM models, *i.e.*, six combinations of protein and ligand descriptors (P0-DLI, P1-DLI, P2-DLI, P0-GD, P1-GD, and P2-GD), six combinations of protein and ligand descriptors with cross-terms, and the only six kinds of cross-terms.

There are a lot of kernel functions used in SVM, such as Normalized Poly Kernel (normalized polynomial kernel), Poly Kernel (polynomial kernel), Precomputed Kernel Matrix Kernel, Puk (Pearson VII function-based universal kernel), RBF Kernel (Radial Basis Function kernel), and String Kernel. Although Poly Kernel and RBF Kernel are most commonly used kernel functions, Puk Kernel is considered as a universal kernel that is capable of serving as a generic alternative to the common linear, polynomial and RBF kernel functions [51]. In fact, we also found that Puk kernel had a stronger mapping power than the other kernels for our data set. For this reason, all models were created using SVR with Puk kernel.

### Validation of PCM models

For each combination of descriptors, 10-fold cross-validation was carried out for the model. The performance of the derived eighteen models was assessed by the goodness-of-fit (R^2^) and predictive ability (Q_cv,_^2^ Q_test_^2^).

Finally, ten known inhibitors [4] were selected as the external validation dataset to assess the specificity performance of the best model. These inhibitors are listed in Table 6 including four pan-HDAC inhibitors (TSA, SAHA, panbinostat, and belinostat), three class I-specific inhibitors (MGCD0103, depsipeptide, and apicidin), and three class II-specific inhibitors (APHA, Tubacin, and NCT-10a). We predicted all the affinity values of the ten inhibitors against all the HDACs with the best model. According to the predicted results, we analyzed the interaction strength of the inhibitors with multiple HDACs and then select out isoform-specific, class-specific as well as pan inhibitors.

**Figure 1:**
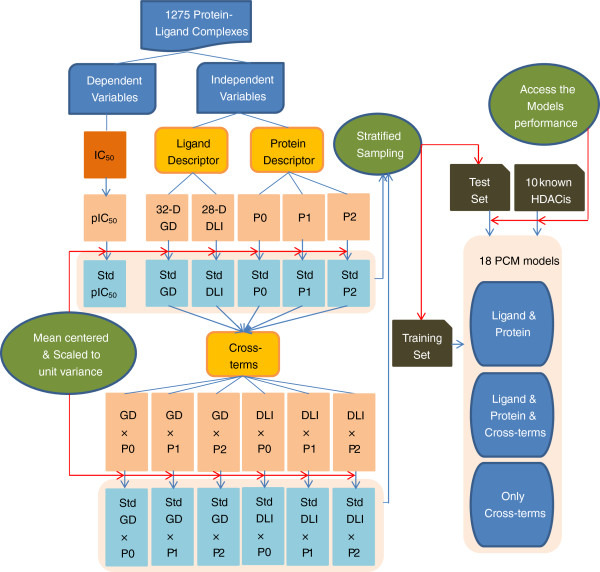
**General framework for our proteochemometric modeling**.

The framework of this work is presented in Figure 1.

## Competing interests

The authors declare that they have no competing interests.

## Authors’ contributions

Conceived and designed the experiments: RZ ZC. Performed the experiments: DW QH YZ QZ. Analyzed the data: DW QH YZ QZ QL JG ZC RZ. Wrote the paper: DW QH YZ QZ QL JG ZC RZ. All authors read and approved the final manuscript.

## Supplementary Material

Additional file 1 **Table S4.** Structures of 1443 chemical compounds (in SMILE format). Click here for file

Additional file 2 **Table S1.** Train set used for construction of the proteochemometric models. Click here for file

Additional file 3 **Table S2.** Test set used for assessment of the proteochemometric models. Click here for file

Additional file 4 **Table S3.** Protein geometry descriptors. Click here for file
