# Supplementary material for: Screening of selective histone deacetylase inhibitors by proteochemometric modeling
Source: BMC Bioinformatics. 2012 Aug 22;13:212. doi: 10.1186/1471-2105-13-212 (PMC3542186; doi:10.1186/1471-2105-13-212)
Supplement: Additional file 1 — Table S4. Structures of 1443 chemical compounds (in SMILE format). [file 1471-2105-13-212-S1.pdf]

Table S1. Train set

HDAC	Ligand	pIC50(μM)	HDAC	Ligand	pIC50(μM)
HDAC2	1	-2.607	HDAC6	294	1.104262
HDAC2	2	-2.81877	HDAC6	295	1.259507
HDAC2	4	1.385853	HDAC6	297	0.934925
HDAC2	5	-1.10396	HDAC6	298	1.01397
HDAC2	6	-1.04027	HDAC6	300	0.853057
HDAC2	9	-0.47415	HDAC6	302	-0.11548
HDAC2	13	1.542366	HDAC6	322	-1.75758
HDAC2	16	-1.33109	HDAC6	323	-1.75758
HDAC2	18	-1.33109	HDAC6	324	-1.75758
HDAC2	19	-1.33109	HDAC6	326	-1.75758
HDAC2	20	-1.33109	HDAC6	327	-1.75758
HDAC2	21	-1.33109	HDAC6	328	-1.75758
HDAC2	22	-1.39764	HDAC6	329	-1.75758
HDAC2	23	-1.33109	HDAC6	331	-1.75758
HDAC2	25	-0.8343	HDAC6	332	-1.75758
HDAC2	26	-1.16742	HDAC6	333	-1.75758
HDAC2	27	-1.33109	HDAC6	335	-1.68315
HDAC2	29	-0.78714	HDAC6	336	-1.63775
HDAC2	31	-0.31215	HDAC6	338	-1.51487
HDAC2	32	-0.36102	HDAC6	339	-1.51204
HDAC2	35	-0.14355	HDAC6	341	-1.35395
HDAC2	36	0.000079	HDAC6	342	-1.3495
HDAC2	38	0.172695	HDAC6	343	-1.20305
HDAC2	40	0.010869	HDAC6	344	-1.13197
HDAC2	42	0.027969	HDAC6	346	-1.06364
HDAC2	43	-0.14355	HDAC6	347	-1.01526
HDAC2	44	0.588367	HDAC6	349	-0.94192
HDAC2	47	0.361983	HDAC6	350	-0.94192
HDAC2	48	0.562687	HDAC6	351	-0.94192
HDAC2	51	0.613188	HDAC6	352	-0.94192
HDAC2	52	0.886033	HDAC6	353	-0.94192
HDAC2	55	0.531864	HDAC6	355	-0.94192
HDAC2	56	0.552914	HDAC6	356	-0.94192
HDAC2	57	0.435104	HDAC6	357	-0.94192
HDAC2	58	0.953095	HDAC6	358	-0.94192
HDAC2	59	0.780137	HDAC6	359	-0.94192
HDAC2	61	0.507728	HDAC6	364	-0.81048
HDAC2	62	0.382231	HDAC6	367	-0.69639
HDAC2	63	0.848032	HDAC6	369	-0.68923
HDAC2	64	1.423176	HDAC6	370	-0.68923
HDAC2	65	0.507728	HDAC6	371	-0.66453

HDAC2	66	0.649243	HDAC6	372	-0.63463
HDAC2	67	0.988351	HDAC6	373	-0.63463
HDAC2	68	0.613188	HDAC6	374	-0.62609
HDAC2	69	0.890379	HDAC6	375	-0.60472
HDAC2	70	0.947913	HDAC6	376	-0.58973
HDAC2	71	0.636812	HDAC6	377	-0.57407
HDAC2	73	0.845271	HDAC6	378	-0.57004
HDAC2	75	1.186175	HDAC6	379	-0.55134
HDAC2	76	1.1467	HDAC6	380	-0.52705
HDAC2	77	1.179047	HDAC6	381	-0.51544
HDAC2	78	1.142856	HDAC6	384	-0.45085
HDAC2	81	1.919917	HDAC6	385	-0.44369
HDAC2	82	1.931533	HDAC6	386	-0.42438
HDAC2	83	1.996118	HDAC6	387	-0.39411
HDAC2	85	-1.75758	HDAC6	388	-0.37882
HDAC2	86	-1.75758	HDAC6	389	-0.37181
HDAC2	87	-1.75758	HDAC6	390	-0.37181
HDAC2	89	-1.75402	HDAC6	392	-0.35364
HDAC2	91	-0.58002	HDAC6	396	-0.31424
HDAC2	92	-0.79473	HDAC6	399	-0.2699
HDAC2	94	1.412105	HDAC6	400	-0.25789
HDAC2	104	-0.06026	HDAC6	401	-0.24546
HDAC2	106	0.805755	HDAC6	403	-0.23258
HDAC2	108	1.259507	HDAC6	404	-0.21921
HDAC2	109	0.443851	HDAC6	406	-0.20531
HDAC2	111	-0.37181	HDAC6	411	-0.16003
HDAC2	114	0.443851	HDAC6	412	-0.16003
HDAC2	120	0.570198	HDAC6	413	-0.1568
HDAC2	121	1.296829	HDAC6	415	-0.12627
HDAC2	123	0.285194	HDAC6	416	-0.12271
HDAC2	124	-0.08099	HDAC6	418	-0.10548
HDAC2	126	-1.44175	HDAC6	419	-0.08895
HDAC2	144	0.522896	HDAC6	421	-0.08499
HDAC2	155	-0.97569	HDAC6	423	-0.08299
HDAC2	170	0.501421	HDAC6	425	-0.07284
HDAC2	177	-0.73272	HDAC6	429	-0.06026
HDAC2	194	-0.33645	HDAC6	432	-0.04277
HDAC2	195	-0.26514	HDAC6	433	-0.02905
HDAC2	197	-0.26514	HDAC6	437	-0.00495
HDAC2	205	0.337544	HDAC6	438	-0.00495
HDAC2	209	0.266459	HDAC6	439	-0.00445
HDAC2	211	0.443851	HDAC6	440	0.000079
HDAC2	212	-0.07694	HDAC6	441	0.010347
HDAC2	213	-0.75502	HDAC6	442	0.015595

HDAC2	214	0.105376	HDAC6	443	0.02633
HDAC2	223	0.079124	HDAC6	445	0.042498
HDAC2	228	1.562614	HDAC6	446	0.05116
HDAC2	232	0.949385	HDAC6	447	0.054684
HDAC2	241	1.104262	HDAC6	449	0.066693
HDAC2	243	0.148806	HDAC6	450	0.066693
HDAC2	244	1.829625	HDAC6	452	0.066693
HDAC2	335	-1.98308	HDAC6	453	0.072854
HDAC2	337	-1.72183	HDAC6	454	0.079124
HDAC2	347	-1.33109	HDAC6	455	0.085507
HDAC2	363	0.956843	HDAC6	456	0.085507
HDAC2	382	-1.11932	HDAC6	457	0.098628
HDAC2	394	-1.74422	HDAC6	458	0.104695
HDAC2	423	-0.59448	HDAC6	459	0.105376
HDAC2	452	-0.95582	HDAC6	460	0.105376
HDAC2	685	-0.94192	HDAC6	462	0.107425
HDAC2	910	0.443851	HDAC6	463	0.112254
HDAC2	1106	0.207283	HDAC6	464	0.119269
HDAC2	1109	0.689388	HDAC6	467	0.131522
HDAC2	1130	0.768433	HDAC6	468	0.13373
HDAC2	1142	-0.08895	HDAC6	469	0.13373
HDAC2	1165	0.87034	HDAC6	470	0.148806
HDAC2	1300	0.655626	HDAC6	471	0.156591
HDAC2	1339	0.980208	HDAC6	473	0.164552
HDAC2	1398	-1.21961	HDAC6	475	0.171873
HDAC2	1399	-1.18746	HDAC6	476	0.172695
HDAC2	1400	-1.01526	HDAC6	477	0.180189
HDAC2	1402	-0.94192	HDAC6	478	0.181031
HDAC2	1403	-0.94192	HDAC6	479	0.189567
HDAC2	1404	-0.79204	HDAC6	480	0.189567
HDAC2	1405	-0.70686	HDAC6	484	0.198314
HDAC2	1406	-0.69639	HDAC6	485	0.201278
HDAC2	1407	-0.69639	HDAC6	486	0.201874
HDAC2	1408	-0.69639	HDAC6	487	0.207283
HDAC2	1410	-0.6511	HDAC6	488	0.207283
HDAC2	1412	-0.50343	HDAC6	490	0.225931
HDAC2	1413	-0.42893	HDAC6	491	0.225931
HDAC2	1416	-0.31424	HDAC6	492	0.235636
HDAC2	1417	-0.19085	HDAC6	493	0.235636
HDAC2	1419	-0.17884	HDAC6	495	0.255884
HDAC2	1420	-0.09731	HDAC6	496	0.255884
HDAC2	1422	-0.0099	HDAC6	498	0.266459
HDAC2	1424	0.060638	HDAC6	500	0.270055
HDAC2	1426	0.119269	HDAC6	501	0.277359

HDAC2	1427	0.119269	HDAC6	502	0.288606
HDAC2	1429	0.119269	HDAC6	504	0.288606
HDAC2	1430	0.119269	HDAC6	505	0.299042
HDAC2	1432	0.119269	HDAC6	506	0.324661
HDAC2	1433	0.119269	HDAC6	509	0.324661
HDAC2	1438	0.156591	HDAC6	510	0.337544
HDAC2	1440	0.266459	HDAC6	511	0.350912
HDAC2	1441	0.277359	HDAC6	516	0.371962
HDAC2	1442	0.288606	HDAC6	517	0.379266
HDAC2	1443	0.410089	HDAC6	518	0.379266
HDAC4	1	-4.34818	HDAC6	519	0.379266
HDAC4	2	-2.66617	HDAC6	520	0.38447
HDAC4	4	-1.51204	HDAC6	521	0.394343
HDAC4	6	-1.00354	HDAC6	522	0.394343
HDAC4	9	-0.37882	HDAC6	524	0.406883
HDAC4	11	0.431665	HDAC6	527	0.436836
HDAC4	12	1.041587	HDAC6	529	0.443851
HDAC4	13	1.780117	HDAC6	531	0.452819
HDAC4	15	-2.03688	HDAC6	532	0.462021
HDAC4	24	-1.14575	HDAC6	534	0.462021
HDAC4	36	0.300221	HDAC6	535	0.462021
HDAC4	79	-0.1852	HDAC6	537	0.481173
HDAC4	80	0.030331	HDAC6	540	0.491152
HDAC4	82	1.804007	HDAC6	541	0.491152
HDAC4	83	1.72994	HDAC6	542	0.501421
HDAC4	92	-0.02436	HDAC6	544	0.501421
HDAC4	94	-1.51204	HDAC6	545	0.501421
HDAC4	95	-1.51204	HDAC6	546	0.511996
HDAC4	96	-1.51204	HDAC6	547	0.511996
HDAC4	101	-1.51204	HDAC6	548	0.522896
HDAC4	102	-0.96423	HDAC6	551	0.545758
HDAC4	144	-0.94192	HDAC6	552	0.58308
HDAC4	220	-0.52427	HDAC6	555	0.624803
HDAC4	228	0.548742	HDAC6	556	0.627768
HDAC4	231	0.048829	HDAC6	557	0.636812
HDAC4	232	1.03214	HDAC6	558	0.655626
HDAC4	240	0.462021	HDAC6	560	0.675494
HDAC4	241	1.140317	HDAC6	563	0.689388
HDAC4	304	-0.94192	HDAC6	564	0.711306
HDAC4	306	-0.94192	HDAC6	566	0.72671
HDAC4	307	-0.94192	HDAC6	567	0.729344
HDAC4	308	-0.65907	HDAC6	568	0.730668
HDAC4	309	-0.16003	HDAC6	571	0.759686
HDAC4	311	0.000079	HDAC6	572	0.777401

HDAC4	312	-0.3555	HDAC6	573	0.81071
HDAC4	314	0.426568	HDAC6	574	0.81071
HDAC4	321	-1.79134	HDAC6	576	0.815735
HDAC4	322	-1.75758	HDAC6	578	0.87034
HDAC4	324	-0.62174	HDAC6	580	0.901163
HDAC4	345	-0.38909	HDAC6	583	0.980208
HDAC4	369	1.259507	HDAC6	586	1.005223
HDAC4	375	-1.34498	HDAC6	589	1.005223
HDAC4	376	0.31223	HDAC6	590	1.022938
HDAC4	385	0.277359	HDAC6	591	1.03214
HDAC4	395	0.635289	HDAC6	592	1.03841
HDAC4	398	-0.34894	HDAC6	593	1.041587
HDAC4	403	-0.2699	HDAC6	595	1.051292
HDAC4	404	-0.26514	HDAC6	597	1.082115
HDAC4	422	0.867986	HDAC6	598	1.093015
HDAC4	425	0.655626	HDAC6	601	1.140317
HDAC4	426	-0.58002	HDAC6	604	1.194922
HDAC4	427	0.815735	HDAC6	607	1.296829
HDAC4	432	1.180461	HDAC6	609	1.361414
HDAC4	433	1.63139	HDAC6	610	1.361414
HDAC4	434	0.696544	HDAC6	612	1.440459
HDAC4	435	-0.03368	HDAC6	614	1.550327
HDAC4	437	0.87034	HDAC6	615	1.602259
HDAC4	439	0.23761	HDAC6	617	1.843165
HDAC4	442	0.707558	HDAC6	621	2.132733
HDAC4	447	0.337544	HDAC7	2	-2.34834
HDAC4	453	0.060638	HDAC7	5	-1.39862
HDAC4	463	0.02633	HDAC7	6	-1.22602
HDAC4	464	0.72671	HDAC7	9	-0.42131
HDAC4	470	0.221176	HDAC7	11	0.682373
HDAC4	476	-0.01479	HDAC7	12	0.65242
HDAC4	480	1.361414	HDAC7	15	-2.26594
HDAC4	485	1.115877	HDAC7	64	0.22307
HDAC4	492	0.700178	HDAC7	79	-0.21729
HDAC4	495	1.440459	HDAC7	80	-0.04092
HDAC4	497	0.181031	HDAC7	81	1.795864
HDAC4	500	1.115877	HDAC7	82	1.668713
HDAC4	503	0.250713	HDAC7	83	1.931533
HDAC4	504	1.209998	HDAC7	92	0.207283
HDAC4	508	1.787903	HDAC7	93	-1.51204
HDAC4	510	0.410089	HDAC7	94	-1.51204
HDAC4	517	0.610343	HDAC7	95	-1.51204
HDAC4	518	1.750581	HDAC7	120	-1.18746
HDAC4	522	0.410089	HDAC7	176	-0.69642

HDAC4	524	1.508604	HDAC7	228	-0.41195
HDAC4	525	0.357791	HDAC7	301	2.320699
HDAC4	532	0.324661	HDAC7	305	-0.94192
HDAC4	533	0.448307	HDAC7	306	-0.94192
HDAC4	542	0.410089	HDAC7	307	-0.94192
HDAC4	543	1.361414	HDAC7	308	-0.68193
HDAC4	550	-0.11548	HDAC7	311	0.164552
HDAC4	554	-0.17884	HDAC7	312	0.245616
HDAC4	555	0.066693	HDAC7	314	0.655626
HDAC4	567	0.717005	HDAC7	315	0.934925
HDAC4	570	0.89478	HDAC8	2	-2.0677
HDAC4	571	-0.63463	HDAC8	3	-2.46175
HDAC4	572	0.596449	HDAC8	4	-1.40538
HDAC4	573	-0.35364	HDAC8	5	0.052917
HDAC4	574	0.815735	HDAC8	6	0.60472
HDAC4	579	2.041401	HDAC8	10	-0.94192
HDAC4	584	1.968855	HDAC8	12	0.333761
HDAC4	592	-0.13674	HDAC8	13	1.153199
HDAC4	594	-0.17268	HDAC8	16	-1.33109
HDAC4	595	1.908671	HDAC8	17	-1.05858
HDAC4	597	1.180461	HDAC8	24	-1.09635
HDAC4	598	1.082115	HDAC8	25	-0.76799
HDAC4	599	1.812343	HDAC8	27	-1.01526
HDAC4	603	1.829625	HDAC8	28	-1.17115
HDAC4	605	-0.06451	HDAC8	31	-0.57805
HDAC4	608	-0.61823	HDAC8	32	-0.56288
HDAC4	609	1.194922	HDAC8	33	-0.58491
HDAC4	614	2.154208	HDAC8	35	-0.49477
HDAC4	622	-2.00312	HDAC8	36	0.300221
HDAC4	624	-1.65207	HDAC8	40	-0.61468
HDAC4	625	-1.55883	HDAC8	42	-0.32247
HDAC4	627	-1.51204	HDAC8	43	-0.56081
HDAC4	629	-1.50691	HDAC8	44	-0.55134
HDAC4	630	-1.4914	HDAC8	46	-0.04589
HDAC4	631	-1.45705	HDAC8	48	-0.53046
HDAC4	633	-1.0324	HDAC8	49	-0.01817
HDAC4	634	-0.95067	HDAC8	50	-0.68923
HDAC4	635	-0.87119	HDAC8	51	-0.04277
HDAC4	636	-0.84842	HDAC8	52	-0.37357
HDAC4	637	-0.84002	HDAC8	56	-0.56576
HDAC4	638	-0.76274	HDAC8	58	-0.36645
HDAC4	639	-0.68923	HDAC8	59	0.084221
HDAC4	640	-0.68851	HDAC8	60	-1.00651
HDAC4	641	-0.64949	HDAC8	62	-0.86745

HDAC4	646	-0.51544	HDAC8	65	-0.1051
HDAC4	649	0.085507	HDAC8	66	0.170233
HDAC4	650	0.092007	HDAC8	67	-0.37003
HDAC4	652	0.624803	HDAC8	68	0.099297
HDAC4	653	1.968855	HDAC8	69	-0.1036
HDAC4	654	2.747188	HDAC8	70	-0.48461
HDAC6	2	-0.76097	HDAC8	71	0.19304
HDAC6	3	-3.1362	HDAC8	72	-0.71164
HDAC6	4	-1.51204	HDAC8	73	0.164552
HDAC6	5	0.711306	HDAC8	74	-0.62522
HDAC6	6	0.491152	HDAC8	75	-1.33109
HDAC6	8	0.296696	HDAC8	76	-0.18789
HDAC6	9	0.613188	HDAC8	77	-0.63293
HDAC6	10	-0.94192	HDAC8	78	-0.22462
HDAC6	11	0.403706	HDAC8	81	-0.39183
HDAC6	13	0.836578	HDAC8	82	-0.29709
HDAC6	14	-1.18746	HDAC8	84	-0.0378
HDAC6	15	-0.46474	HDAC8	85	-1.75758
HDAC6	17	-0.37357	HDAC8	87	-1.75758
HDAC6	18	-1.33109	HDAC8	93	-1.51204
HDAC6	21	0.043069	HDAC8	94	-1.51204
HDAC6	22	-0.02905	HDAC8	95	-1.51204
HDAC6	23	-0.31631	HDAC8	96	-1.51204
HDAC6	24	-1.35579	HDAC8	97	-1.51204
HDAC6	25	0.706441	HDAC8	100	-1.37197
HDAC6	26	0.172695	HDAC8	103	-1.23697
HDAC6	27	0.416589	HDAC8	104	-1.23697
HDAC6	28	0.132257	HDAC8	105	-1.22122
HDAC6	29	0.642973	HDAC8	108	-1.18746
HDAC6	30	1.831401	HDAC8	110	-1.18746
HDAC6	32	0.804773	HDAC8	111	-1.18746
HDAC6	33	1.097471	HDAC8	113	-1.18746
HDAC6	34	0.92791	HDAC8	114	-1.18746
HDAC6	35	1.060261	HDAC8	115	-1.12401
HDAC6	36	1.505044	HDAC8	116	-1.06111
HDAC6	40	1.765041	HDAC8	117	-1.01149
HDAC6	41	0.968333	HDAC8	118	-0.97729
HDAC6	42	1.066369	HDAC8	123	-0.94192
HDAC6	43	0.682373	HDAC8	126	-0.93113
HDAC6	44	0.938485	HDAC8	127	-0.93113
HDAC6	46	2.093333	HDAC8	128	-0.87206
HDAC6	49	1.812343	HDAC8	129	-0.83238
HDAC6	50	1.641138	HDAC8	131	-0.8176
HDAC6	51	1.919917	HDAC8	132	-0.81558

HDAC6	52	1.616525	HDAC8	134	-0.80427
HDAC6	53	1.876927	HDAC8	135	-0.80006
HDAC6	55	1.606951	HDAC8	136	-0.799
HDAC6	57	1.542366	HDAC8	143	-0.7034
HDAC6	58	2.227761	HDAC8	145	-0.69639
HDAC6	59	1.982224	HDAC8	146	-0.69067
HDAC6	60	1.566806	HDAC8	147	-0.67822
HDAC6	66	1.897771	HDAC8	148	-0.67672
HDAC6	67	1.919917	HDAC8	149	-0.66531
HDAC6	68	1.847796	HDAC8	151	-0.65271
HDAC6	70	1.804007	HDAC8	152	-0.63882
HDAC6	72	1.943542	HDAC8	154	-0.62349
HDAC6	73	2.093333	HDAC8	156	-0.61734
HDAC6	76	1.897771	HDAC8	157	-0.61646
HDAC6	81	1.876927	HDAC8	163	-0.57034
HDAC6	82	1.996118	HDAC8	165	-0.55873
HDAC6	83	1.887196	HDAC8	167	-0.5416
HDAC6	84	2.075162	HDAC8	168	-0.5383
HDAC6	87	-1.75758	HDAC8	169	-0.51779
HDAC6	88	-1.25205	HDAC8	170	-0.51544
HDAC6	89	-1.06111	HDAC8	171	-0.491
HDAC6	90	-0.73653	HDAC8	172	-0.47942
HDAC6	91	-0.16003	HDAC8	173	-0.47415
HDAC6	92	-0.94192	HDAC8	174	-0.47415
HDAC6	93	-1.51204	HDAC8	177	-0.42742
HDAC6	94	-1.51204	HDAC8	178	-0.42438
HDAC6	96	-1.51204	HDAC8	179	-0.42131
HDAC6	101	-1.51204	HDAC8	184	-0.32853
HDAC6	102	-1.26223	HDAC8	187	-0.27645
HDAC6	104	0.277359	HDAC8	188	-0.24546
HDAC6	105	-0.64296	HDAC8	191	-0.24036
HDAC6	106	-1.51204	HDAC8	192	-0.21647
HDAC6	107	-1.75758	HDAC8	193	-0.20332
HDAC6	108	-1.18746	HDAC8	194	-0.19379
HDAC6	110	-1.18746	HDAC8	195	-0.18789
HDAC6	115	-1.51204	HDAC8	196	-0.18188
HDAC6	120	-1.18746	HDAC8	200	-0.12627
HDAC6	121	-2.43662	HDAC8	203	-0.07694
HDAC6	122	-0.94192	HDAC8	204	-0.05812
HDAC6	123	-0.94192	HDAC8	205	-0.04722
HDAC6	126	-1.33109	HDAC8	207	-0.03825
HDAC6	127	-0.94192	HDAC8	210	0.000079
HDAC6	132	0.054684	HDAC8	211	0.005176
HDAC6	137	0.655626	HDAC8	212	0.005176

HDAC6	139	-0.94192	HDAC8	213	0.02633
HDAC6	144	0.624803	HDAC8	214	0.031822
HDAC6	145	1.412105	HDAC8	217	0.050575
HDAC6	163	0.06976	HDAC8	218	0.066693
HDAC6	170	1.412105	HDAC8	219	0.066693
HDAC6	176	0.255884	HDAC8	220	0.098628
HDAC6	177	0.501421	HDAC8	224	0.119269
HDAC6	179	-0.42285	HDAC8	225	0.164552
HDAC6	187	-0.00793	HDAC8	226	0.181031
HDAC6	189	-0.15353	HDAC8	228	0.198314
HDAC6	191	-0.6511	HDAC8	229	0.198314
HDAC6	194	0.655626	HDAC8	232	0.231722
HDAC6	200	-0.30793	HDAC8	233	0.268612
HDAC6	204	-0.53864	HDAC8	234	0.288606
HDAC6	208	-0.09286	HDAC8	237	0.364806
HDAC6	211	-0.03825	HDAC8	238	0.379266
HDAC6	212	1.01397	HDAC8	240	0.394343
HDAC6	213	-0.52705	HDAC8	243	0.426568
HDAC6	215	-0.35677	HDAC8	244	0.443851
HDAC6	217	-0.24546	HDAC8	245	0.443851
HDAC6	221	-0.74896	HDAC8	246	0.443851
HDAC6	222	1.505044	HDAC8	248	0.522896
HDAC6	223	0.545758	HDAC8	249	0.522896
HDAC6	224	0.198314	HDAC8	250	0.522896
HDAC6	225	0.522896	HDAC8	251	0.522896
HDAC6	226	1.01397	HDAC8	252	0.545758
HDAC6	228	1.571048	HDAC8	253	0.588367
HDAC6	229	0.037401	HDAC8	255	0.624803
HDAC6	230	0.522896	HDAC8	256	0.624803
HDAC6	231	-0.25544	HDAC8	257	0.624803
HDAC6	232	0.655626	HDAC8	258	0.689388
HDAC6	234	0.443851	HDAC8	261	0.689388
HDAC6	235	-1.54581	HDAC8	262	0.689388
HDAC6	237	0.934925	HDAC8	263	0.689388
HDAC6	238	1.022938	HDAC8	264	0.772889
HDAC6	240	0.481173	HDAC8	265	0.772889
HDAC6	242	-0.01416	HDAC8	266	0.772889
HDAC6	244	0.972247	HDAC8	267	0.772889
HDAC6	247	0.689388	HDAC8	269	0.853057
HDAC6	250	0.844721	HDAC8	270	0.853057
HDAC6	251	1.01397	HDAC8	272	0.853057
HDAC6	252	-0.94192	HDAC8	273	0.853057
HDAC6	253	1.104262	HDAC8	275	0.934925
HDAC6	257	1.180461	HDAC8	277	0.934925

HDAC6	260	0.624803	HDAC8	278	0.934925
HDAC6	261	0.772889	HDAC8	279	0.934925
HDAC6	265	0.853057	HDAC8	280	0.934925
HDAC6	266	0.853057	HDAC8	282	0.934925
HDAC6	268	0.689388	HDAC8	283	0.964461
HDAC6	269	0.772889	HDAC8	284	1.01397
HDAC6	270	0.772889	HDAC8	285	1.01397
HDAC6	271	0.853057	HDAC8	286	1.01397
HDAC6	273	0.853057	HDAC8	287	1.01397
HDAC6	274	0.485131	HDAC8	288	1.07154
HDAC6	275	0.689388	HDAC8	290	1.104262
HDAC6	276	0.689388	HDAC8	291	1.104262
HDAC6	277	0.772889	HDAC8	292	1.104262
HDAC6	279	0.772889	HDAC8	293	1.180461
HDAC6	280	0.853057	HDAC8	294	1.180461
HDAC6	282	1.180461	HDAC8	295	1.180461
HDAC6	283	-0.03916	HDAC8	296	1.259507
HDAC6	284	0.689388	HDAC8	298	1.259507
HDAC6	285	0.689388	HDAC8	300	1.338552
HDAC6	286	0.853057	HDAC8	301	2.320699
HDAC6	290	0.689388			
