# Supplementary material for: Screening of selective histone deacetylase inhibitors by proteochemometric modeling
Source: BMC Bioinformatics. 2012 Aug 22;13:212. doi: 10.1186/1471-2105-13-212 (PMC3542186; doi:10.1186/1471-2105-13-212)
Supplement: Additional file 2 — Table S1. Train set used for construction of the proteochemometric models. [file 1471-2105-13-212-S2.pdf]

Table S2. Test set

HDAC	Ligand	pIC50(μM)	HDAC	Ligand	pIC50(μM)
HDAC2	3	0.89478	HDAC6	321	-0.64378
HDAC2	7	1.005223	HDAC6	325	-0.65271
HDAC2	8	0.972247	HDAC6	330	-0.66992
HDAC2	10	1.694359	HDAC6	334	-0.69082
HDAC2	11	2.256114	HDAC6	337	-0.62087
HDAC2	12	2.320699	HDAC6	340	-0.61739
HDAC2	14	0.89478	HDAC6	345	-0.57407
HDAC2	15	0.907662	HDAC6	348	-0.56699
HDAC2	17	0.964461	HDAC6	354	-0.56081
HDAC2	24	1.505044	HDAC6	360	-0.51544
HDAC2	28	1.051292	HDAC6	361	-0.51307
HDAC2	30	1.093015	HDAC6	362	-0.49727
HDAC2	33	1.127886	HDAC6	363	-0.491
HDAC2	34	1.346953	HDAC6	365	-0.45925
HDAC2	39	1.750581	HDAC6	366	-0.42438
HDAC2	41	1.338552	HDAC6	368	-0.40717
HDAC2	45	1.743566	HDAC6	382	-0.38909
HDAC2	46	1.505044	HDAC6	383	-0.38655
HDAC2	49	1.716818	HDAC6	391	-0.37678
HDAC2	50	1.795864	HDAC6	393	-0.37181
HDAC2	60	1.993295	HDAC6	394	-0.37181
HDAC2	72	2.154208	HDAC6	395	-0.35177
HDAC2	74	1.838594	HDAC6	397	-0.3461
HDAC2	84	4.52213	HDAC6	398	-0.33448
HDAC2	88	0.934925	HDAC6	402	-0.32652
HDAC2	90	0.934925	HDAC6	405	-0.3245
HDAC2	93	1.723318	HDAC6	407	-0.32247
HDAC2	95	1.931533	HDAC6	408	-0.31627
HDAC2	96	1.857242	HDAC6	409	-0.31838
HDAC2	101	2.041401	HDAC6	410	-0.30366
HDAC2	103	0.988351	HDAC6	417	-0.30151
HDAC2	105	0.972685	HDAC6	420	-0.29276
HDAC2	110	1.919917	HDAC6	422	-0.28151
HDAC2	112	1.134047	HDAC6	424	-0.27575
HDAC2	113	1.132806	HDAC6	426	-0.24546
HDAC2	115	1.519504	HDAC6	427	-0.25544
HDAC2	133	0.988351	HDAC6	428	-0.2699
HDAC2	137	1.209998	HDAC6	430	-0.24546
HDAC2	145	1.606951	HDAC6	431	-0.24546
HDAC2	171	1.01397	HDAC6	434	-0.24546
HDAC2	176	1.857242	HDAC6	435	-0.21647

HDAC2	208	1.153199	HDAC6	436	-0.24292
HDAC2	220	2.399745	HDAC6	444	-0.19671
HDAC2	222	1.542366	HDAC6	448	-0.19085
HDAC2	225	1.061271	HDAC6	451	-0.19085
HDAC2	226	1.412105	HDAC6	461	-0.19085
HDAC2	237	1.127886	HDAC6	465	-0.16641
HDAC2	301	2.093333	HDAC6	466	-0.17578
HDAC2	303	2.010578	HDAC6	472	-0.14691
HDAC2	321	1.082115	HDAC6	474	-0.14355
HDAC2	344	0.934925	HDAC6	481	-0.12414
HDAC2	360	1.723318	HDAC6	482	-0.13328
HDAC2	432	2.320699	HDAC6	483	-0.13845
HDAC2	686	1.01397	HDAC6	494	-0.11875
HDAC2	698	1.03214	HDAC6	497	-0.11181
HDAC2	801	1.298803	HDAC6	499	-0.08895
HDAC2	1131	1.955972	HDAC6	503	-0.08895
HDAC2	1132	1.990494	HDAC6	507	-0.08379
HDAC2	1312	1.931533	HDAC6	508	-0.08499
HDAC2	1397	0.914284	HDAC6	512	-0.08139
HDAC2	1401	1.005223	HDAC6	513	-0.06451
HDAC2	1409	1.051292	HDAC6	514	-0.06451
HDAC2	1411	1.093015	HDAC6	515	-0.06451
HDAC2	1414	1.115877	HDAC6	523	-0.05597
HDAC2	1415	1.127886	HDAC6	525	-0.04554
HDAC2	1418	1.127886	HDAC6	526	-0.04722
HDAC2	1421	1.166568	HDAC6	528	-0.03825
HDAC2	1423	1.194922	HDAC6	530	-0.02436
HDAC2	1425	1.259507	HDAC6	533	-0.01479
HDAC2	1428	1.259507	HDAC6	536	-0.01479
HDAC2	1431	1.259507	HDAC6	538	-0.01479
HDAC2	1434	1.220407	HDAC6	539	-0.00793
HDAC2	1435	1.209998	HDAC6	543	-0.00694
HDAC2	1436	1.361414	HDAC6	549	0.020386
HDAC2	1437	1.406697	HDAC6	550	0.043069
HDAC2	1439	1.505044	HDAC6	553	0.043069
HDAC4	3	0.443851	HDAC6	554	0.049992
HDAC4	5	0.501421	HDAC6	559	0.098628
HDAC4	7	0.491152	HDAC6	561	0.105376
HDAC4	8	0.493325	HDAC6	562	0.106057
HDAC4	10	0.545758	HDAC6	565	0.112254
HDAC4	14	0.447411	HDAC6	569	0.112254
HDAC4	45	0.624803	HDAC6	570	0.119269
HDAC4	64	0.853057	HDAC6	575	0.14612
HDAC4	81	0.87034	HDAC6	577	0.148806

HDAC4	84	0.876294	HDAC6	579	0.189567
HDAC4	93	0.483147	HDAC6	581	0.189567
HDAC4	124	0.522896	HDAC6	582	0.193913
HDAC4	176	0.630757	HDAC6	584	0.249687
HDAC4	244	0.772889	HDAC6	585	0.225931
HDAC4	287	0.853057	HDAC6	587	0.266459
HDAC4	301	0.876294	HDAC6	588	0.261132
HDAC4	302	0.624803	HDAC6	594	0.288606
HDAC4	303	0.522896	HDAC6	596	0.288606
HDAC4	305	0.514149	HDAC6	599	0.300221
HDAC4	310	0.689388	HDAC6	600	0.28956
HDAC4	313	0.672105	HDAC6	602	0.31223
HDAC4	315	0.772889	HDAC6	603	0.324661
HDAC4	323	0.462021	HDAC6	605	0.324661
HDAC4	346	0.689388	HDAC6	606	0.324661
HDAC4	348	0.772889	HDAC6	608	0.350912
HDAC4	363	0.730668	HDAC6	611	0.350912
HDAC4	377	0.682373	HDAC6	613	0.364806
HDAC4	381	0.772889	HDAC6	616	0.392805
HDAC4	383	0.593735	HDAC6	618	0.418232
HDAC4	389	0.591041	HDAC6	619	0.416589
HDAC4	390	0.655626	HDAC6	620	0.426568
HDAC4	416	0.772889	HDAC7	3	-0.8614
HDAC4	440	0.815735	HDAC7	4	-0.85391
HDAC4	441	0.772889	HDAC7	7	-0.81558
HDAC4	443	0.81071	HDAC7	8	-0.83133
HDAC4	444	0.681304	HDAC7	10	-0.79473
HDAC4	445	0.815735	HDAC7	13	-0.7428
HDAC4	449	0.791295	HDAC7	24	-0.83495
HDAC4	450	0.81071	HDAC7	36	-0.75382
HDAC4	455	0.665421	HDAC7	45	-0.77826
HDAC4	456	0.76932	HDAC7	106	-0.8221
HDAC4	459	0.841986	HDAC7	121	-0.79473
HDAC4	465	0.689388	HDAC7	244	-0.76391
HDAC4	479	0.624803	HDAC7	302	-0.80531
HDAC4	481	0.859296	HDAC7	303	-0.80796
HDAC4	490	0.772889	HDAC7	304	-0.81456
HDAC4	498	0.87034	HDAC7	309	-0.74957
HDAC4	502	0.689388	HDAC7	310	-0.74713
HDAC4	506	0.815735	HDAC8	1	-4.09789
HDAC4	507	0.853057	HDAC8	7	-1.18746
HDAC4	520	0.853057	HDAC8	8	-0.94192
HDAC4	521	0.759686	HDAC8	9	-0.94192
HDAC4	523	0.853057	HDAC8	11	-0.91622

HDAC4	546	0.772889	HDAC8	14	-3.12889
HDAC4	548	0.742815	HDAC8	15	-3.08159
HDAC4	562	0.772889	HDAC8	21	-1.90822
HDAC4	581	0.826003	HDAC8	29	-1.75758
HDAC4	586	0.853057	HDAC8	30	-1.61039
HDAC4	604	0.873304	HDAC8	41	-1.11062
HDAC4	612	0.87034	HDAC8	45	-1.18746
HDAC4	623	0.479211	HDAC8	47	-1.75758
HDAC4	626	0.481173	HDAC8	53	-0.94192
HDAC4	628	0.481173	HDAC8	55	-1.2804
HDAC4	642	0.624803	HDAC8	61	-1.30284
HDAC4	643	0.624803	HDAC8	63	-1.35104
HDAC4	644	0.655626	HDAC8	64	-0.94192
HDAC4	645	0.646094	HDAC8	83	-1.33109
HDAC4	647	0.689388	HDAC8	86	-2.18051
HDAC4	651	0.722796	HDAC8	88	-2.28242
HDAC6	7	-0.20531	HDAC8	89	-2.38633
HDAC6	12	0.198314	HDAC8	90	-2.39228
HDAC6	16	-0.58973	HDAC8	91	-3.05534
HDAC6	19	-0.59353	HDAC8	92	-2.13838
HDAC6	20	-0.5973	HDAC8	98	-2.03688
HDAC6	31	0.177256	HDAC8	99	-1.98681
HDAC6	38	0.148806	HDAC8	101	-1.96579
HDAC6	39	0.119269	HDAC8	102	-1.79295
HDAC6	45	0.098628	HDAC8	106	-1.75758
HDAC6	47	0.337544	HDAC8	107	-1.75758
HDAC6	48	0.394343	HDAC8	109	-1.75758
HDAC6	56	0.374866	HDAC8	112	-1.7611
HDAC6	61	0.379266	HDAC8	119	-1.75758
HDAC6	62	0.443851	HDAC8	120	-1.75758
HDAC6	63	0.364806	HDAC8	121	-1.75758
HDAC6	64	0.386724	HDAC8	122	-1.75758
HDAC6	65	0.371962	HDAC8	124	-1.75758
HDAC6	69	0.426568	HDAC8	125	-1.75758
HDAC6	71	0.426568	HDAC8	130	-1.75758
HDAC6	74	0.410089	HDAC8	133	-1.75758
HDAC6	75	0.443851	HDAC8	137	-1.75758
HDAC6	77	0.414953	HDAC8	138	-1.75758
HDAC6	78	0.443851	HDAC8	139	-1.7025
HDAC6	85	-0.62867	HDAC8	140	-1.68728
HDAC6	86	-0.63463	HDAC8	141	-1.59949
HDAC6	95	-0.61734	HDAC8	142	-1.51613
HDAC6	103	-0.60103	HDAC8	144	-1.51204
HDAC6	109	-0.58198	HDAC8	150	-1.51204

HDAC6	116	-0.63463	HDAC8	153	-1.51204
HDAC6	131	-0.51544	HDAC8	155	-1.51204
HDAC6	133	-0.38909	HDAC8	158	-1.51204
HDAC6	135	-0.51544	HDAC8	159	-1.51204
HDAC6	146	-0.52648	HDAC8	160	-1.45993
HDAC6	171	-0.45085	HDAC8	161	-1.45447
HDAC6	173	-0.40557	HDAC8	162	-1.42221
HDAC6	181	-0.5492	HDAC8	164	-1.37229
HDAC6	188	-0.55347	HDAC8	166	-1.37124
HDAC6	193	-0.21647	HDAC8	175	-1.33109
HDAC6	195	-0.08776	HDAC8	176	-1.33109
HDAC6	197	0.288606	HDAC8	180	-1.33109
HDAC6	203	-0.48846	HDAC8	181	-1.33109
HDAC6	205	-0.19484	HDAC8	182	-1.33109
HDAC6	206	-0.31424	HDAC8	183	-1.33109
HDAC6	207	-0.45092	HDAC8	185	-1.30155
HDAC6	209	0.364806	HDAC8	186	-1.18746
HDAC6	210	-0.62609	HDAC8	189	-1.18746
HDAC6	214	0.360114	HDAC8	190	-1.18746
HDAC6	218	-0.36825	HDAC8	197	-1.18746
HDAC6	220	-0.491	HDAC8	198	-1.16929
HDAC6	241	0.098628	HDAC8	199	-1.08555
HDAC6	243	-0.05424	HDAC8	202	-1.03213
HDAC6	245	0.031822	HDAC8	206	-0.98834
HDAC6	246	0.106741	HDAC8	208	-0.97569
HDAC6	248	0.141942	HDAC8	209	-0.97569
HDAC6	249	0.126425	HDAC8	215	-0.96089
HDAC6	254	0.126425	HDAC8	216	-0.94894
HDAC6	255	0.124982	HDAC8	221	-0.94192
HDAC6	256	0.119269	HDAC8	222	-0.94192
HDAC6	258	-0.18493	HDAC8	223	-0.94192
HDAC6	259	0.066693	HDAC8	227	-0.94192
HDAC6	262	0.119269	HDAC8	230	-0.94192
HDAC6	263	0.267534	HDAC8	231	-0.94192
HDAC6	264	0.054684	HDAC8	235	-0.94192
HDAC6	272	0.176839	HDAC8	236	-0.94192
HDAC6	278	0.119269	HDAC8	239	-0.94192
HDAC6	281	0.189567	HDAC8	241	-0.94192
HDAC6	287	0.350912	HDAC8	242	-0.94192
HDAC6	288	0.307377	HDAC8	247	-0.94192
HDAC6	291	0.119269	HDAC8	254	-0.94192
HDAC6	292	0.166166	HDAC8	259	-0.94192
HDAC6	293	0.119269	HDAC8	260	-0.94192
HDAC6	296	0.158169	HDAC8	268	-0.94192

HDAC6	299	0.156591	HDAC8	271	-0.94192
HDAC6	301	0.356405	HDAC8	274	-0.94192
HDAC6	316	-0.73463	HDAC8	276	-0.92001
HDAC6	317	-0.73015	HDAC8	281	-0.92746
HDAC6	318	-0.72102	HDAC8	289	-0.92001
HDAC6	319	-0.69639	HDAC8	297	-0.89086
HDAC6	320	-0.69639	HDAC8	299	-0.86288
