# Supplementary material for: Screening of selective histone deacetylase inhibitors by proteochemometric modeling
Source: BMC Bioinformatics. 2012 Aug 22;13:212. doi: 10.1186/1471-2105-13-212 (PMC3542186; doi:10.1186/1471-2105-13-212)
Supplement: Additional file 3 — Table S2. Test set used for assessment of the proteochemometric models. [file 1471-2105-13-212-S3.pdf]

Table S3 Protein Geometry descriptors

	C-N-CA	C-N-CA(G)	C-N-CA(P)	CA-C-N	CA-C-N(G)	CA-C-N(P)	CA-C-O	CA-C-O(G)	CB-CA-C	CB-CA-C(A)
HDAC2	121.65	121.67	120.71	116.67	116.68	116.73	120.38	120.39	110.20	110.54
HDAC4	122.19	122.09	121.41	116.59	116.59	116.81	120.35	120.40	110.09	110.34
HDAC6	121.67	121.87	120.18	116.47	116.70	116.68	120.34	120.46	110.36	110.68
HDAC7	121.85	121.40	121.50	116.55	116.74	117.28	120.40	120.47	110.43	110.55
HDAC8	122.23	122.13	121.78	116.43	116.24	116.56	120.41	120.42	110.46	110.52

Table S3 (continued)

	CB-CA-C (I,T,V)	N-CA-C	N-CA-C (G)	N-CA-C (P)	N-CA-CB	N-CA-CB (A)	N-CA-CB (I,T,V)	N-CA-CB (P)	O-C-N	O-C-N (P)
HDAC2	110.62	111.30	112.97	112.17	110.70	110.91	111.70	103.20	122.91	123.15
HDAC4	109.92	111.42	113.56	113.48	110.54	110.57	111.67	103.25	123.01	123.39
HDAC6	110.73	111.70	113.84	113.61	110.86	110.43	112.22	103.33	123.12	123.21
HDAC7	110.83	111.48	113.42	113.32	110.71	110.40	112.09	103.16	122.96	122.87
HDAC8	110.79	111.43	113.01	112.63	110.65	110.55	111.45	103.34	123.13	123.66

Table S3 (continued)

	Chi1 g(+)	Chi1 g(-)	Chi1 trans	Omega	Phi	Phi helix	Phi(P)	Psi	Psi helix	Psi(G)
HDAC2	-65.77	56.29	182.91	179.28	-76.25	-64.07	-71.37	85.02	-38.10	28.12
HDAC4	-64.92	52.52	183.40	180.22	-79.18	-61.03	-65.83	80.73	-36.27	8.74
HDAC6	-63.51	55.77	182.95	179.23	-75.12	-70.89	-69.64	73.57	-33.24	-21.09
HDAC7	-65.86	55.78	182.78	179.14	-74.80	-60.81	-69.90	80.70	-37.93	21.27
HDAC8	-66.64	51.01	185.75	179.07	-76.79	-62.42	-69.48	78.32	-40.28	20.39
